# Glycine triggers a non-ionotropic activity of GluN2A-containing NMDA receptors to confer neuroprotection

**DOI:** 10.1038/srep34459

**Published:** 2016-10-03

**Authors:** Rong Hu, Juan Chen, Brendan Lujan, Ruixue Lei, Mi Zhang, Zefen Wang, Mingxia Liao, Zhiqiang Li, Yu Wan, Fang Liu, Hua Feng, Qi Wan

**Affiliations:** 1 Department of Physiology and Cell Biology, University of Nevada School of Medicine, 1664 North Virginia Street, MS0352, Reno, Nevada, 89557, USA; 2 Department of Neurosurgery, Southwest Hospital, Third Military Medical University, Chongqing 400038, China; 3 Department of Physiology, School of Basic Medical Sciences, Wuhan University School of Medicine, 185 Donghu Street, Wuhan 430071, China; 4 Department of Neurology, the Central Hospital of Wuhan, Wuhan 430060, China; 5 Department of Neurosurgery, Zhongnan Hospital, Wuhan University School of Medicine, 169 Donghu Street, Wuhan 430071, China; 6 Campbell Research Institute, Centre for Addiction and Mental Health, and Departments of Psychiatry, University of Toronto, 250 College Street, Toronto, Ontario, M5T 1R8, Canada

## Abstract

Ionotropic activation of NMDA receptors (NMDARs) requires agonist glutamate and co-agonist glycine. Here we show that glycine enhances the activation of cell survival-promoting kinase Akt in cultured cortical neurons in which both the channel activity of NMDARs and the glycine receptors are pre-inhibited. The effect of glycine is reduced by shRNA-mediated knockdown of GluN2A subunit-containing NMDARs (GluN2ARs), suggesting that a non-ionotropic activity of GluN2ARs mediates glycine-induced Akt activation. In support of this finding, glycine enhances Akt activation in HEK293 cells over-expressing GluN2ARs. The effect of glycine on Akt activation is sensitive to the antagonist of glycine-GluN1 binding site. As a functional consequence, glycine protects against excitotoxicity-induced neuronal death through the non-ionotropic activity of GluN2ARs and the neuroprotective effect is attenuated by Akt inhibition. Thus, this study reveals an unexpected role of glycine in eliciting a non-ionotropic activity of GluN2ARs to confer neuroprotection via Akt activation.

The N-methyl-D-aspartate receptor (NMDAR) is a subtype of ionotropic glutamate receptors that mediate the vast majority of excitatory neurotransmission in the mammalian central nervous system (CNS)^1^. NMDARs are ligand-gated Ca^2+^-permeable channels that consist of GluN1, GluN2 (GluN2A-GluN2D) and GluN3 (GluN3A-GluN3B) subunits^2^. The GluN2A- and GluN2B-containing NMDARs (GluN2ARs and GluN2BRs) are the major combinations of NMDARs expressed in CNS^1^. The binding of agonist glutamate to GluN2 subunits and co-agonist glycine to GluN1 subunits is required to activate GluN2ARs and GluN2BRs^3^, which play essential roles in synaptic plasticity^4^,^5^, neural development^6^,^7^ and glutamate-induced neurotoxicity^8^,^9^,^10^.

Different GluN2 subunits confer distinct roles of NMDAR subtypes and link them with different intracellular signaling pathways^11^,^12^,^13^. Previous evidence suggests that GluN2BR-mediated neurotoxicity induces neuronal death^14^,^15^,^16^, and that enhancement of GluN2AR activity promotes neuronal survival^16^,^17^,^18^. However, the molecular mechanisms underlying the differential effects of GluN2ARs and GluN2BRs in neuronal survival and death are not fully understood.

While it is well known for its ionotropic function, NMDAR has been recently shown to have non-ionotropic activity^19^,^20^,^21^,^22^,^23^,^24^,^25^. For example, ligand binding to NMDARs is sufficient to induce long-term depression (LTD), but does not require ion flow through NMDARs^20^. A non-ionotropic activity is found to be mediated through GluN2BR and is required for β-amyloid–induced synaptic depression^21^,^22^. The non-ionotropic activity of NMDARs is shown to drive structural shrinkage at spiny synapses^24^ and couple Src family kinases to pannexin-1 in excitotoxic injury^25^. In the present study, we reveal that glycine alone elicits a non-ionotropic activity of GluN2ARs but not GluN2BRs. We demonstrate that glycine confers neuroprotection through non-ionotropic activation of GluN2ARs and subsequent enhancement of Akt activation.

## Results

### Glycine increases Akt phosphorylation independent of Ca^2+^ influx through NMDAR channels

**Figure 1:**
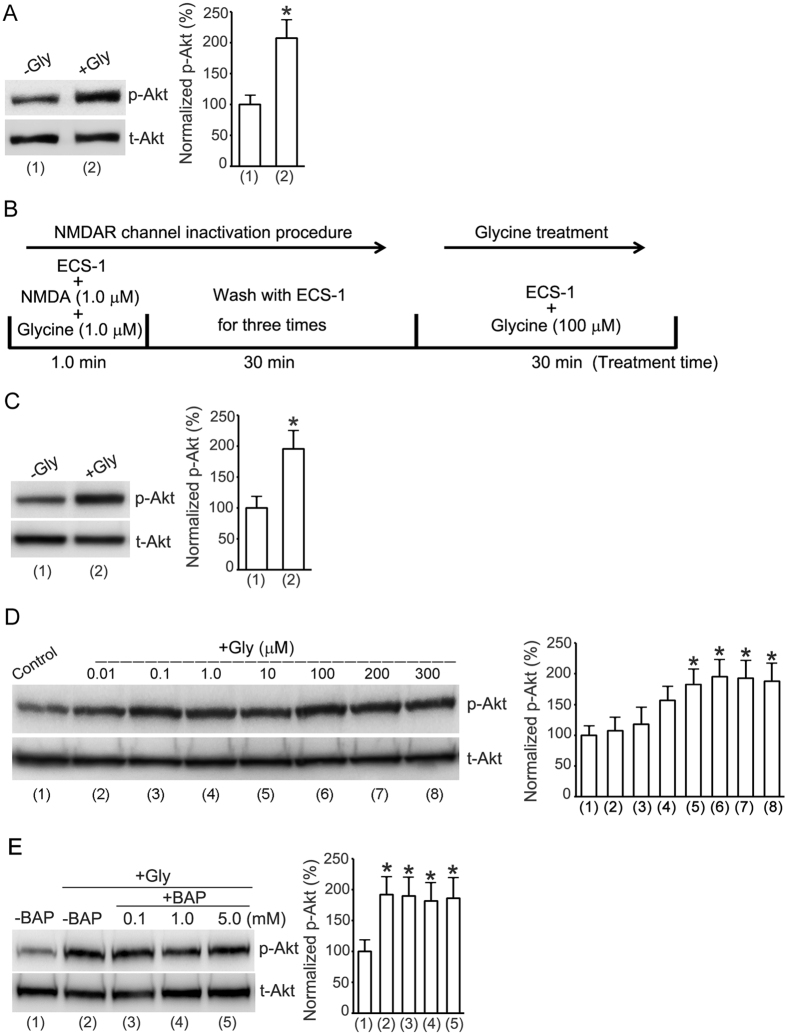
Enhancement of Akt phosphorylation by glycine in cortical neurons does not require Ca^2+^-mediated channel activities of NMDARs. (**A**) Glycine (100 μM) increases Akt phosphorylation (p-Akt) in neurons treated with ECS without addition of Ca^2+^ but with addition of 5.0 mM EGTA (n = 7, Student’s *t* test, *p < 0.05 vs. −Gly). (**B**) A schematic diagram showing the NMDAR channel inactivation and glycine treatment procedure. (**C**) Glycine (100 μM) increases p-Akt in neurons where NMDAR channel activities are inhibited (n = 9, Student’s *t* test, *p < 0.05 vs. −Gly). (**D**) Glycine-induced increase of p-Akt is dose-dependent in neurons where NMDAR channel activities are inhibited (n = 6, ANOVA test, *p < 0.05 vs. control). (**E**) The enhancement of p-Akt by glycine (100 μM) is not altered by BAPTA that is included in the ECS-1 (n = 6, ANOVA test, *p < 0.05 vs. -BAP). The p-Akt analyses were normalized to group (1) labeled in the bar graphs. Gly: glycine; BAP: BAPTA.

To test the effect of glycine on the activation of cell survival-promoting kinase Akt (protein kinase B) in cultured mouse cortical neurons where no Ca^2+^ pass through the NMDAR channels, we treated the neurons with glycine (100 μM) for 30 min in a modified extracellular solution (5.0 mM EGTA, 137 mM NaCl, 5.4 mM KCl, 1.0 mM MgCl_2_, 25 mM HEPES, 33 mM Glucose, titrated to pH 7.4 with osmolarity of 300–320 mOsm) in which Ca^2+^ was not included but with the addition of 5.0 mM EGTA to chelate the residual Ca^2+^. The activation of Akt was quantified by measuring Akt phosphorylation (p-Akt) on Ser473 in western blot assay^26^,^27^. The levels of p-Akt were quantified by calculating the ratio of p-Akt to total Akt (t-Akt). Our results showed that treatment of glycine (100 μM) for 30 min increased Akt phosphorylation in the cortical neurons in which there were no Ca^2+^ influx into the NMDAR channels (Fig. 1A).

To exclude the possibility that residual Ca^2+^ in the extracellular solution might pass through NMDAR channels, we treated the neurons with the extracellular solution described above but with the addition of non-competitive NMDAR antagonist MK-801^28^,^29^. We named this specific Ca^2+^-free extracellular solution as ECS-1 (10 μM MK-801, 5.0 mM EGTA, 137 mM NaCl, 5.4 mM KCl, 1.0 mM MgCl_2_, 25 mM HEPES, 33 mM Glucose, titrated to pH 7.4 with osmolarity of 300–320 mOsm). Since MK-801 is a use-dependent pharmacological agent, we first treated the neurons with NMDA (1.0 μM) and glycine (1.0 μM) for 1.0 min that opened the NMDAR channels and allowed MK-801 in the ECS-1 to fully block NMDARs^28^,^29^,^30^. The cultured neurons were then washed with ECS-1 for three times (10 min wash/each). As this procedure eliminates the possibility of Ca^2+^ in activation of NMDAR channels, in this study we referred to this as NMDAR channel inactivation procedure (Fig. 1B). As shown in Fig. 1C, glycine (100 μM) treatment increased Akt phosphorylation in the cortical neurons where the NMDAR channel activity was inhibited by the NMDAR channel inactivation procedure. In the same experimental conditions, the effect of glycine on Akt phosphorylation was found to be dose-dependent (Fig. 1D). These data indicate the possibility that glycine-induced Akt activation is independent of the channel activity of NMDARs.

To provide further evidence that the effect of glycine on Akt phosphorylation was independent of extracellular Ca^2+^, we tested the effect of BAPTA, a Ca^2+^ chelator that has faster calcium-binding kinetics than EGTA^31^. The experimental condition was same as that in Fig. 1C. But BAPTA (0.1, 1.0 or 5.0 mM) was included in the ECS-1 in the +BAP groups (Fig. 1E). BAPTA was included both during the NMDAR channel inactivation procedure and during treatment with glycine. Compared with the group without BAPTA treatment, BAPTA treatment did not interfere with glycine-induced elevation of Akt phosphorylation (Fig. 1E), suggesting that Ca^2+^-mediated channel activity of NMDARs does not contribute to the observed effect of glycine in cortical neurons.

### Elevation of Akt phosphorylation by glycine does not depend on the activation of glycine receptors

**Figure 2:**
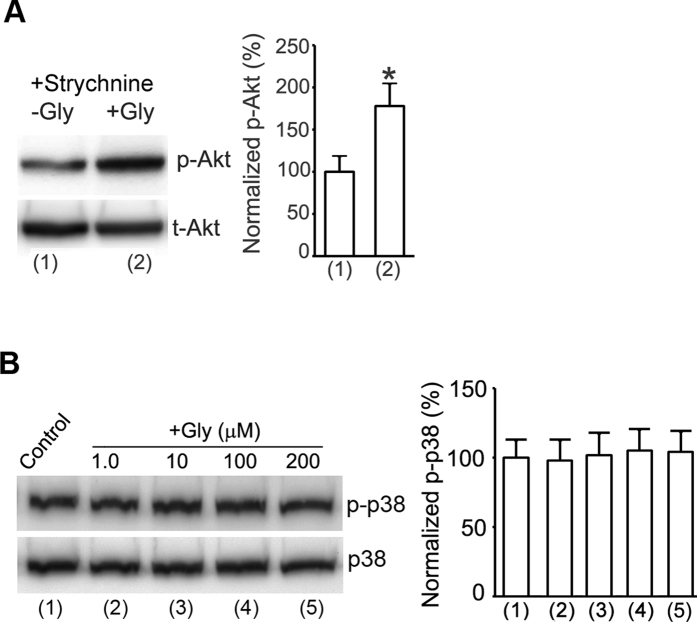
Enhancement of Akt phosphorylation by glycine in cortical neurons does not require activation of glycine receptors. (**A**) Treatment of strychnine (10 μM) does not interfere with the enhancement of p-Akt by glycine (100 μM) in neurons where NMDAR channel activities are pre-inhibited (n = 6, Student’s *t* test, *p < 0.05 vs. −Gly). (**B**) Glycine has no significant effect on p38-MAPK phosphorylation (p-p38) in cortical neurons following NMDAR channel inactivation procedure (n = 6; ANOVA test). Gly: glycine; p38: p38-MAPK.

Glycine is the agonist for strychnine-sensitive glycine receptors. Glycine receptors are not significantly expressed in the mature but expressed in the developing cortex^32^,^33^. To exclude the possible effect of glycine receptors on the observed Akt activation by glycine, we used the same experimental design as that in Fig. 1C. But strychnine was added into the ECS-1 for all the treatment in both –Gly and +Gly group (Fig. 2A). Our data showed that strychnine (10 μM) failed to block the enhancement of Akt phosphorylation by glycine (100 μM) in cortical neurons subjected to NMDAR channel inactivation procedure (Fig. 2A). Thus, glycine-induced enhancement of Akt phosphorylation does not depend on the activation of strychnine-sensitive glycine receptors.

### Glycine does not affect p38-MAPK activation in cortical neurons where NMDAR channel activities and glycine receptors are inhibited

The p38-MAPK is implicated in NMDAR-dependent LTD^34^, and was shown to be activated by non-ionotropic NMDAR signaling after chemical LTD induction^20^,^24^. The activation of p38-MAPK is also involved in excitotoxicity^35^. To determine whether glycine also altered p38-MAPK signaling independent of glycine receptors and the activation of NMDAR channels, we tested the effect of glycine on p38-MAPK phosphorylation in cortical neurons following the experimental procedure described in Fig. 2A. Our results showed that glycine (100 μM) had no significant effect on p38-MAPK phosphorylation in our experimental conditions (Fig. 2B), suggesting that a specific activation of Akt but not p38-MAPK by glycine occurs in the condition in which NMDAR channel activities and glycine receptors were suppressed.

### Glycine enhances Akt activation through a non-ionotropic activation of GluN2ARs

**Figure 3:**
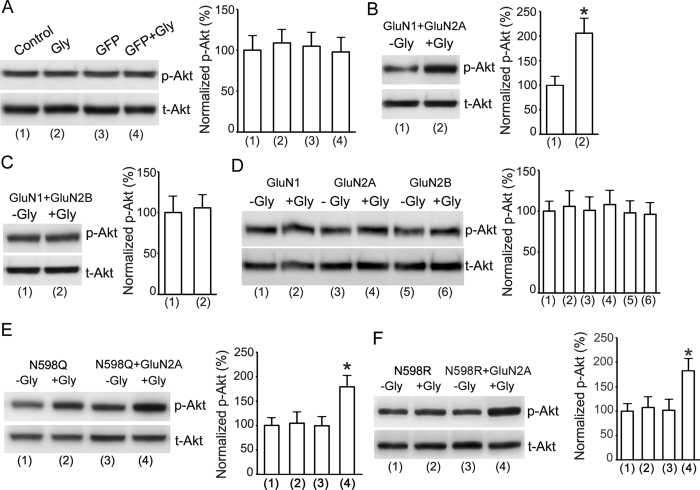
Non-ionotropic activity of GluN2AR mediates glycine-induced enhancement of Akt phosphorylation in HEK293 cells expressing GluN2ARs. (**A**) In HEK293 cells without or with GFP transfection, the levels of p-Akt are not altered by glycine (100 μM) treatment after the channel activities of NMDARs are inhibited by the NMDAR channel inactivation procedure (n = 6; ANOVA test). (**B**) In HEK293 cells transfected with GluN1 + GluN2A cDNAs, glycine (100 μM) increases p-Akt after the channel activities of NMDARs are inhibited (n = 9, Student’s *t* test, *P < 0.05 vs. -Gly). (**C**) In HEK293 cells transfected with GluN1 + GluN2B cDNAs, the levels of p-Akt are not altered by glycine (100 μM) after the channel activities of NMDARs are inhibited (n = 6; Student’s *t* test). (**D**) In HEK293 cells transfected with GluN1, GluN2A or GluN2B cDNAs, respectively, the levels of p-Akt are not altered by glycine (100 μM) after the channel activities of NMDARs are inhibited (n = 6; ANOVA test). (**E**) In HEK293 cells transfected with GluN1(N598Q) + GluN2A, but not GluN1(N598Q) alone, glycine enhances Akt phosphorylation after the channel activities of NMDARs are inhibited (n = 6, ANOVA test, *P < 0.05 vs. -Gly). (**F**) Glycine increases Akt phosphorylation in HEK293 cells transfected with GluN1(N598R) + GluN2A following NMDAR channel inactivation procedure (n = 6; ANOVA test, *P < 0.05 vs. -Gly). Gly: glycine.

Our results thus far suggest a non-ionotropic activity of NMDAR to mediate the potentiation of Akt activation by glycine. To provide direct evidence for this possibility, we measured the effects of glycine on Akt phosphorylation in HEK293 cells transiently over-expressing NMDARs. The cDNAs of GluN1, GluN2A and/or GluN2B subunits were transfected in various combinations into the HEK293 cells^36^. The Ca^2+^-mediated channel activities of NMDARs expressed in the transfected cells were inhibited by the NMDAR channel inactivation procedure. Treatment of glycine (100 μM) for 30 min had no effect on Akt phosphorylation in both non-transfected HEK293 cells and the cells transfected with cDNAs of green fluorescence protein (GFP) (Fig. 3A). However, glycine increased Akt phosphorylation in HEK293 cells transfected with cDNAs of GluN1 + GluN2A following the NMDAR channel inactivation procedure (Fig. 3B), but not in cells transfected with cDNAs of GluN1 + GluN2B (Fig. 3C). We also found that glycine did not increase Akt phosphorylation in HEK293 cells transfected with cDNAs of GluN1, GluN2A and GluN2B, respectively (Fig. 3D). Together, these results indicate that a non-ionotropic activity of GluN2ARs mediates the elevation of Akt phosphorylation by glycine.

The N598Q and N598R in GluN1 subunit is a critical residue at the selectivity filter of the NMDAR channel that determines calcium permeability^37^. The GluN1(N598Q) mutant and GluN1(N598R) mutant have been shown to cause decreased calcium permeability of NMDAR channels^37^,^38^,^39^. To test the effect of GluN1(N598Q) and GluN1(N598R) on the enhancement of Akt activation by glycine, we transfected GluN1(N598Q), GluN2A + GluN1(N598Q), GluN1(N598R), GluN2A + GluN1(N598R) in HEK293 cells that were treated with standard ECS (137 mM NaCl, 5.4 mM KCl, 1.3 mM CaCl_2_, 1.0 mM MgCl_2_, 25 mM HEPES, 33 mM Glucose, titrated to pH 7.4 with osmolarity of 300–320 mOsm). As shown in Fig. 3E,F, glycine (100 μM) increased Akt phosphorylation in HEK293 cells transfected with GluN2A + GluN1(N598Q) or GluN2A + GluN1(N598R). These results provide molecular evidence supporting the notion that GluN2AR-mediated Akt activation is independent of Ca^2+^ influx.

**Figure 4:**
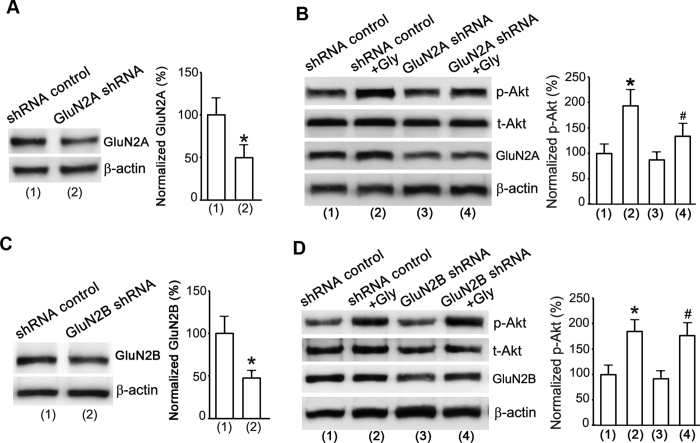
Non-ionotropic activity of GluN2AR mediates glycine-induced enhancement of Akt phosphorylation in cortical neurons. (**A**) The GluN2A protein expression in cortical neurons is suppressed by GluN2A shRNA (n = 6, Student’s *t* test, *P < 0.05 vs. shRNA control). (**B**) GluN2A knockdown by GluN2A shRNA attenuates glycine-induced increase of p-Akt in cortical neurons where NMDAR channels are inhibited (n = 6, ANOVA test, *P < 0.05 vs. shRNA control; ^#^*P* < 0.05 vs. shRNA control + Gly). (**C**) The GluN2B protein expression in cortical neurons is suppressed by GluN2B shRNA transduction (n = 6, Student’s *t* test, *P < 0.05 vs. shRNA control). (**D**) GluN2B knockdown by GluN2B shRNA does not interfere with glycine-induced increase of p-Akt in cortical neurons where NMDAR channels activities are inhibited (n = 6, ANOVA test, *P < 0.05 vs. shRNA control; ^#^*P* < 0.05 vs. GluN2B shRNA). Gly: glycine.

To validate the role of a non-ionotropic activity of GluN2AR in mediating the enhancement of Akt activation by glycine in cortical neurons, we applied a GluN2A knockdown approach. The GluN2A protein expression was suppressed in the cultured cortical neurons transducted with GluN2A shRNA lentiviral particles (Fig. 4A). The same experimental design as that in Fig. 1C was applied to inhibit NMDARs. As shown in Fig. 4B, glycine (100 μM) increased Akt phosphorylation in neurons transducted with lentiviral shRNA control, but the effect of glycine was significantly reduced in neurons transducted with lentiviral GluN2A shRNA. As another control of GluN2A shRNA against GluN2A, the GluN2B shRNA had no influence on the observed effect of glycine (Fig. 4C,D). These results lead us to conclude that glycine promotes Akt activation through a non-ionotropic activity of GluN2ARs in cortical neurons.

### The glycine-GluN1 binding site mediates the non-ionotropic activation of GluN2ARs

**Figure 5:**
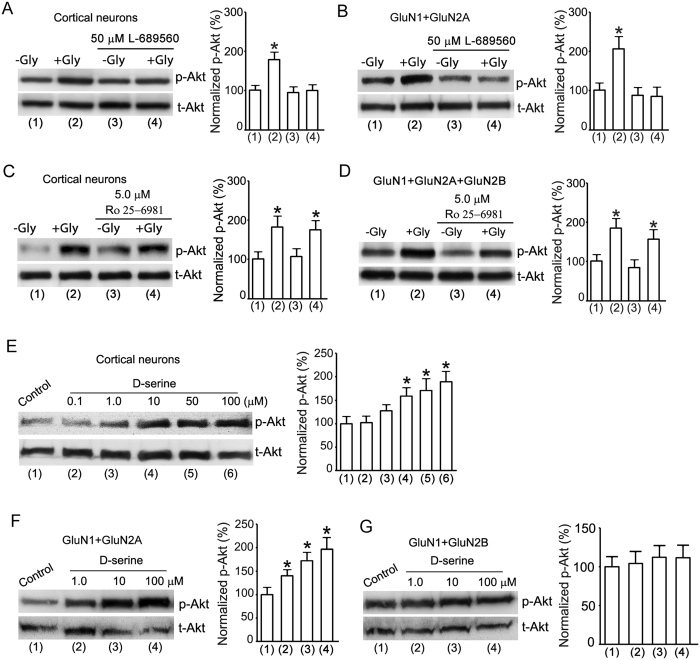
The glycine-GluN1 binding is required for glycine-induced non-ionotropic activation of GluN2ARs. (**A**) Glycine-GluN1 binding site antagonist L-689560 (50 μM) blocks glycine (100 μM)-induced increase of p-Akt in cultured cortical neurons after the channel activities of NMDARs are inhibited (n = 6, ANOVA test, *P < 0.05 vs.-Gly). (**B**) L-689560 (50 μM) blocks glycine (100 μM)-induced increase of p-Akt in HEK293 cells transfected with GluN1 + GluN2A after the channel activities of NMDARs are inhibited (n = 6, ANOVA test, *P < 0.05 vs.-Gly). (**C**) GluN2BR antagonist Ro 25-6981 (5.0 μM) does not interfere with glycine (100 μM)-induced increase of p-Akt in cultured cortical neurons following the NMDAR channel inactivation procedure (n = 6, ANOVA test, *P < 0.05 vs.-Gly). (**D**) Ro 25-6981 (5.0 μM) does not interfere with glycine (100 μM)-induced increase of p-Akt in HEK293 cells transfected with GluN1 + GluN2A + GluN2B following the NMDAR channel inactivation procedure (n = 6, ANOVA test, *P < 0.05 vs.-Gly). (**E**) D-serine increases the level of p-Akt in cultured cortical neurons after the channel activities of NMDARs are inhibited (n = 6, ANOVA test, *P < 0.05 vs. Control). (**F**) D-serine increases the level of p-Akt in HEK293 cells transfected with GluN1 + GluN2A after the channel activities of NMDARs are inhibited (n = 5, ANOVA test, *P < 0.05 vs. Control). **(G**) D-serine has no effect on the level of p-Akt in HEK293 cells transfected with GluN1 + GluN2B after the channel activities of NMDARs are inhibited (n = 5, ANOVA test). Gly: glycine.

To determine how glycine exerts its effect through the non-ionotropic activation of GluN2ARs, we tested the effect of glycine-GluN1 binding site antagonist L-689560 on glycine-induced Akt activation after Ca^2+^-mediated channel activities of NMDARs were inhibited^40^,^41^,^42^,^43^. The L-689560 was included in the ECS-1 in the wash step of the NMDAR channel inactivation procedure and in the step of glycine treatment (Fig. 1B). The cultures were then treated with ECS-1 containing glycine (100 μM) and L-689560 for 30 min. We showed that after the channel activities of NMDARs were inhibited, L-689560 (50 μM) blocked glycine-induced Akt phosphorylation in the cultured neurons and in the HEK293 cells transfected with GluN1 + GluN2A (Fig. 5A,B). These data suggest that the glycine-GluN1 binding is required for the non-ionotropic activation of GluN2ARs.

As a control study, we also tested the effect GluN2B antagonist Ro 25-6981 (5.0 μM)^42^,^43^. Our data showed that Ro 25-6981 had no significant effect on glycine-induced Akt activation in the neurons and the HEK293 cells transfected with GluN1 + GluN2A + GluN2B after Ca^2+^-mediated NMDAR channel activity was inhibited (Fig. 5C,D).

D-serine is the endogenous agonist of glycine-GluN1 binding site^44^. As a further support for the role of glycine-GluN1 binding in mediating the effect of non-ionotropic GluN2ARs, we tested the role of D-serine in Akt phosphorylation in the cortical neurons and the HEK293 cells transfected with GluN1 + GluN2A or GluN1 + GluN2B following NMDAR channel inactivation procedure. D-serine increased Akt phosphorylation in both cortical neurons and HEK293 cells transfected with cDNAs of GluN1 + GluN2A but not with those of GluN1 + GluN2B (Fig. 5E–G).

### Glycine prevents glutamate neurotoxicity-induced neuronal death through non-ionotropic activation of GluN2ARs

**Figure 6:**
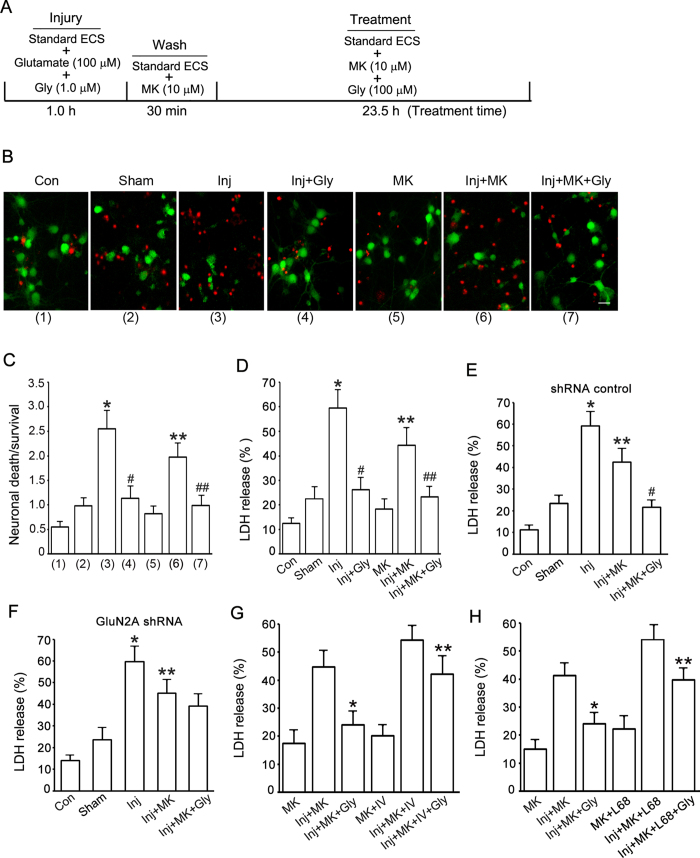
Glycine protects against glutamate neurotoxicity-induced neuronal injury in cortical neurons through non-ionotropic activation of GluN2ARs. (**A**) A schematic diagram showing glutamate neurotoxicity injury and glycine treatment procedure. (**B**) Representative images showing that glycine (100 μM) reduces glutamate neurotoxicity-induced cell death in neurons where NMDAR channel activity is inactivated. Green: FDA; Red: PI. Scale bar = 25 μm. (**C**) Summarized data of B (n = 5. Total 3136 cells counted for Con group, 2825 cells for Sham group, 3225 cells for Inj group, 3208 cells for Inj + Gly group, 3003 cells for MK group, 3160 cells for Inj + MK group and 3231 cells for Inj + MK + Gly group. ANOVA test, *P < 0.05 vs. Sham; ^#^*P* < 0.05 vs. Inj; **P < 0.05 vs. Inj; ^##^*P* < 0.05 vs. Inj + MK). (**D**) In neurons where NMDAR channel activities are inhibited, glycine (100 μM) prevents glutamate neurotoxicity-induced increase of LDH release (n = 6, ANOVA test, *P < 0.05 vs. Sham; ^#^*P* < 0.05 vs. Inj; **P < 0.05 vs. Inj; ^##^*P* < 0.05 vs. Inj + MK). (**E**) Glycine (100 μM) reduces glutamate neurotoxicity-induced increase of LDH release in neurons where shRNA control is transfected and NMDAR channel activity is suppressed (n = 6, ANOVA test, *P < 0.05 vs. Sham; **P < 0.05 vs. Inj; ^#^*P* < 0.05 vs. Inj + MK). (**F**) Glycine (100 μM) does not prevent glutamate neurotoxicity-induced increase of LDH release in neurons where GluN2A expression is suppressed by GluN2A shRNA and NMDAR channel activity is inhibited (n = 6, ANOVA test, *P < 0.05 vs. Sham; **P < 0.05 vs. Inj). (**G**) Akt inhibitor IV (1.0 μM) decreases glycine (100 μM)-induced reduction of LDH release in neurons where NMDAR channel activity is inhibited (n = 6, ANOVA test, *P < 0.05 vs. Inj + MK; **P < 0.05 vs. Inj + MK + Gly). (**H**) Glycine-GluN1 binding antagonist L-689560 (50 μM) decreases glycine (100 μM)-induced reduction of LDH release in neurons where NMDAR channel activity is inhibited (n = 6, ANOVA test, *P < 0.05 vs. Inj + MK; **P < 0.05 vs. Inj + MK + Gly). Con: control; Inj: injury; Gly: glycine; MK: MK-801; L68: L-689560.

As Akt is a survival-promoting kinase that plays a crucial role in preventing neuronal death^26^,^45^,^46^, we measured the effect of non-ionotropic activation of NMDARs by glycine on Akt phosphorylation in glutamate neurotoxicity-induced neuronal injury. The injury was produced by treating the cultured cortical neurons with standard ECS containing glutamate (100 μM) and glycine (1.0 μM) for 1.0 h (Fig. 6A). To block Ca^2+^-mediated channel activities of NMDARs, following 1.0 h injury and 30 min wash with standard ECS, the cultures were treated with standard ECS containing 10 μM MK-801 for 23.5 h. For the Control group (Con; Fig. 6B–F), the culture was treated with maintenance medium for 25 h. For the Sham group (Fig. 6B–F), the culture was treated with standard ECS for 25 h. For the injury group (Inj; Fig. 6B–F), the cultures were treated with standard ECS for 24 h following the injury by glutamate (100 μM) + glycine (1.0 μM) for 1.0 h. For the group of glycine, MK-801 or MK-801 + glycine treatment in injured cultures (Inj + Gly, Inj + MK or Inj + MK + Gly; Fig. 6B–D), following the 1.0 h injury the cultures were first washed with standard ECS containing MK-801 (10 μM) for three times (10 min wash/each), and then treated with standard ECS containing glycine (100 μM), MK-801 (10 μM) or MK-801 (10 μM) + glycine (100 μM) for 23.5 h. For the group of MK-801 treatment in uninjured cultures (MK; Fig. 6B–D), the cultures were treated with standard ECS containing MK-801 (10 μM) for 25 h. The Double labeling of propidium iodide (PI) and fluorescein diacetate (FDA) was performed to measure neuronal viability^47^. The levels of lactate dehydrogenase (LDH) released from injured neurons was also measured to quantify the neuronal damage^27^. Our data showed that after glutamate neurotoxicity insult, glycine (100 μM) treatment protected against the death of cortical neurons in which Ca^2+^-mediated NMDAR channel activity was inhibited (Fig. 6B–D).

Our results further demonstrated that the neuroprotective effect of glycine (100 μM) was reduced in injured cortical neurons in which the GluN2A expression was suppressed by lentiviral GluN2A shRNA (Fig. 6E,F). The neurons in both shRNA control and GluN2A shRNA groups were subjected to the same experimental procedures described in Fig. 6B–D. We conclude that the neuroprotective effect of glycine is at least in part mediated through non-ionotropic activation of GluN2ARs in glutamate neurotoxicity-induced neuronal injury.

To determine the roles of Akt activation and glycine-GluN1 binding in glycine-induced neuroprotection, we tested the effect of Akt inhibitor IV and glycine-GluN1 binding antagonist L-689560 in our experimental model. The experimental condition was the same as that described in Fig. 6A–D. The Akt inhibitor IV and L-689560 were included in both wash and treatment steps. We found that both Akt inhibitor IV (1.0 μM) and L-689560 (50 μM) significantly reduced glycine-induced neuroprotective effect in neurons where Ca^2+^-mediated channel activity of NMDARs were inhibited (Fig. 6G,H). Thus, Akt activation and glycine-GluN1 binding mediate glycine-induced neuroprotection. Together, these results provide functional evidence for the role of non-ionotropic activity of GluN2ARs in mediating the neuroprotective effect of glycine.

## Discussion

Using a Ca^2+^-free ECS-based procedure to inactivate the channel activity of NMDARs in cultured cortical neurons and HEK293 cells expressing GluN2ARs, we tested the effect of glycine on Akt phosphorylation, a cellular process playing important role in neuronal survival. We provided the first evidence that glycine induced a potentiation of Akt phosphorylation independent of the channel activity of NMDARs. We confirmed that glycine-induced non-ionotropic activation of GluN2ARs, but not GluN2BRs, mediated the enhancement of Akt activation. Thus, our study identified a non-ionotropic function of GluN2ARs. To ensure no Ca^2+^-mediated channel activities of NMDARs contributing to glycine-induced effect in our study, we established a NMDAR channel inactivation procedure in which Ca^2+^ was not included in the ECS but with the addition of Ca^2+^ chelator EGTA and the use-dependent open channel blocker MK-801. To allow MK-801 to fully block the channels of NMDARs, we pretreated the cells with NMDA and glycine to open the NMDAR channels. Thus, Ca^2+^ influx through NMDARs would not likely occur in our experimental conditions.

Increasing evidence supports the non-ionotropic function of NMDARs^19^,^20^,^21^,^22^,^23^,^24^,^25^. It has been recently shown that a non-ionotropic activation of NMDAR was insensitive to the glycine-GluN1 site antagonist^20^,^21^,^24^. However, our study shows that the glycine-GluN1 binding is required to activate the non-ionotropic activity of GluN2ARs. These findings suggest that glycine triggers a non-ionotropic activity of GluN2ARs through the glycine-GluN1 binding site. By testing the effects of glycine in HEK293 transfected with different combinations of NMDARs, we were able to obtain direct evidence to reveal that a non-ionotropic activation of GluN2ARs but not GluN2BRs mediates the enhancement of Akt activation.

The novel non-ionotropic NMDAR may have functional significance. NMDAR-mediated neurotoxicity induces neuronal death and neurodegeneration in various CNS disorders including ischemic stroke, traumatic brain injury and neurodegenerative diseases^48^,^49^,^50^. However, the use of NMDAR antagonists as neuroprotective agents was disappointing in clinical trials^51^,^52^,^53^. A simple possibility is that these antagonists, while suppressing NMDAR-mediated neurotoxicity, block the biological and/or neural survival-promoting effects of NMDARs^1^,^16^,^17^,^18^. Thus, identification of molecular mechanisms by which specific NMDAR subtype selectively exerts its effect on neuronal survival or death would provide a critical basis for the development of potent therapy for CNS injuries and neurodegenerative diseases.

GluN2ARs and GluN2BRs play different role in neuronal survival or death^14^,^16^. But the underlying molecular mechanism remains unclear. It is recently reported that a non-ionotropic function of NMDARs was required for β-amyloid–induced synaptic depression and synaptic loss^21^,^22^,^23^, providing new evidence for the involvement of GluN2BRs in neurotoxicity. Our observation for a non-ionotropic activation of GluN2ARs by glycine explains in part why GluN2AR plays a different role than GluN2BR in neuronal survival.

The molecular mechanism underlying glycine-induced non-ionotropic NMDAR activation is unclear. It is possible that glycine binds to GluN1 and induces conformational changes in GluN2A. If this is the case, why it cannot change the conformation of GluN2B? The different structure between GluN1-GluN2A coupling and GluN1-GLuN2B coupling may be the possible reason. Future study is required to reveal the mechanisms.

How non-ionotropic activity of GluN2ARs enhances Akt activation is unclear. It is likely that the C-terminal domain of GluN2A may mediate the effect of glycine on Akt activation. Akt deactivation is believed to be a causal mediator of cell death^26^,^45^,^46^. Enhancement of Akt activity exerts pro-survival effect in neuronal injury and neurodegenerative diseases^26^,^45^,^46^. In this study, we identify Akt as a downstream neuroprotective signal of glycine that activates non-ionotropic GluN2ARs. We provide evidence that non-ionotropic activation of GluN2ARs by glycine reduces glutamate neurotoxicity-induced Akt deactivation and thus prevents cortical neuronal death. Akt is known to influence neuronal survival through activation or inhibition of substrates^26^,^45^,^46^. For example, activated Akt promotes survival through phosphorylation of transcription factors forkhead/FOXO, NF-κB and mdm2 or through phosphorylation of Bcl-2 family members Bad and Bim^26^,^45^,^46^. Further study is needed to determine which Akt-dependent signal pathway mediates the activation of non-ionotropic GluN2ARs by glycine.

It would be important to determine where is glycine coming from considering the brain does not normally have glycinergic transmissions. Glial cells would be the source of glycine release, especially during the ischemia injury process. In this study, we show that D-serine has similar effect to glycine, suggesting that D-serine is also the endogenous agonist for this non-ionotropic NMDA receptor activity.

## Experimental Procedures

### General methods

Randomization was used to assign samples to the experimental groups, and to collect and process data. All animal experiments were approved and carried out in compliance with the IACUC guidelines of University of Nevada and Wuhan University School of Medicine. All experimental protocols were approved by the Animal Care and Ethics Committee of University of Nevada and Wuhan University School of Medicine.

### Neuronal culture

The cortical neuronal cultures were prepared from female C57BL/6 mice at gestation day 17 as described^27^,^54^. Briefly, dissociated neurons were suspended in plating medium (Neurobasal medium, 2% B-27 supplement, 0.5% FBS, 0.5 μM L-glutamine, and 25 μM glutamic acid) and plated on poly-D-lysine coated Petri dishes. After 1 day in culture, half of the plating medium was removed and replaced with maintenance medium (Neurobasal medium, 2% B-27 supplement, and 0.5 μM L-glutamine). Thereafter, maintenance medium was changed in the same manner every 3 days. The cultured neurons were used for experiments at 12 days after plating.

### HEK293 cell culture, plasmid transfections and shRNA lentiviral particle treatment

HEK293 cells were grown in RPMI 1640 medium (Life Technologies, Grand Island, NY) supplemented with 10% FBS and Pen/Strep (10 μg/ml). The plasmids of GFP, GluN1, GluN2A, GluN2B, GluN1(N598Q), GluN1(N598R) were transfected in cultured HEK293 cells^36^. Transfections were performed using Lipofectamine 2000 (Invitrogen, Carlsbad, CA) as described in our previous studies^15^,^27^. DNA-Lipofectamine complexes were made in serum-free medium Opti-MEM. To prevent NMDAR-induced cell death, the transfected HEK293 cells were treated with 1.0 mM DAPV to prevent NMDAR-induced excitotoxicity^55^. The transduction of GluN2A and GluN2B shRNA lentiviral particles were performed in cultured cortical neurons based on the manufacturer’s instructions.

### Western blotting

Western blotting assay was performed as described previously^15^,^27^. For the detection of phospho-Akt, the samples prepared in the same day were used. The polyvinylidene difluoride membrane (Millipore, Bedford, MA, USA) was incubated with primary antibody against phospho-Akt (Ser473) (Cell Signaling Technology, Beverly, MA), Akt (Cell Signaling Technology, Beverly, MA), phospho-p38-MAPK (Cell Signaling Technology, Beverly, MA), p38-MAPK (Cell Signaling Technology, Beverly, MA), β-actin (Santa Cruz Biotech), GluN2A (Santa Cruz Biotech), or GluN2B (Santa Cruz Biotech). Primary antibodies were labeled with horseradish peroxidase-conjugated secondary antibody, and protein bands were imaged using SuperSignal West Femto Maximum Sensitivity Substrate (Pierce, Rockford, IL, USA). The EC3 Imaging System (UVP, LLC, Upland, CA) was used to obtained blot images directly from the polyvinylidene difluoride membrane. For the detection of total Akt, the same polyvinylidene difluoride membrane was stripped and then re-incubated with primary antibody against total Akt (Cell Signaling Technology). The quantification of Western blot data was performed using ImageJ software.

### Neuronal viability assays

Double staining of propidium iodide (PI) and fluorescein diacetate (FDA) was performed to detect neuronal viability using a modified procedure^47^. Briefly, cultures were rinsed with extracellular solution and incubated with FDA (5 μM) and PI (2 μM) for 30 min. The cultures were washed with extracellular solution and then viewed on an Olympus fluorescent microscope (IX51, Olympus). Neuronal viability was determined by calculating the number of PI-labeled cells over FDA-labeled cells. The investigator for the cell count was blinded to the experimental treatment.

The lactate dehydrogenase (LDH) is a cytoplasmic enzyme retained by viable cells with intact plasma membranes and released from cells with damaged membranes. The LDH release was measured using CytoTox 96 Cytotoxicity kit based on the manufacturer’s instructions (Promega, Madison, WI)^27^. The levels of maximal LDH release were measured by treating the cultures with 10× lysis solution (provided by the manufacturer) to yield complete lysis of the cells. Absorbance data were obtained using a 96-well plate reader (Molecular Devices, Palo Alto, CA) at 490 nm. According to the manufacturer’s instructions, the LDH release (%) was calculated by calculating the ratio of experimental LDH release to maximal LDH release.

### Statistics

Student’s *t* test or ANOVA test was used where appropriate to examine the statistical significance of the differences between groups of data. Newman–Keuls tests were used for post-hoc comparisons when appropriate. All results are presented as mean ± SE. Significance was placed at *p* < 0.05.

## Additional Information

**How to cite this article**: Hu, R. *et al*. Glycine triggers a non-ionotropic activity of GluN2A-containing NMDA receptors to confer neuroprotection. *Sci. Rep.* **6**, 34459; doi: 10.1038/srep34459 (2016).
